# Acute Melancholia

**Published:** 1905-04-01

**Authors:** 


### ACUTE MELANCHOLIA.

The causation of psychoses which have no known organic basis is naturally enough a fruitful source of speculation. In this field of learning there are, broadly speaking, two schools—one believing the condition to be an expression of disordered metabolism, and in essence a secondary evidence of toxic absorption, the other crediting the symptoms to some unknown primary perversion of the mind. Dr. A. A. D. Townsend[1] is a partially convinced disciple of the toxic school, and has brought forward some interesting data in support of his position.

Dealing with acute melancholia, he says that the sufferers always present certain features pointing to an intoxication of some kind—namely, a foul breath, a furred tongue, and some degree of anæmia; in addition, they lack appetite and are constipated. Moreover, these appearances are always associated with an excess in the urine of indoxyl. This substance, when present in the urine in excess, is an indication of abnormal intestinal putrefaction. Indol is a product of albuminous digestion, and on entering the blood is oxidised to indoxyl; this again combines with potassium sulphate to form indoxyl potassium sulphate, in which form it is eliminated in the urine. This body is normally present, but in infinitesimal quantity. The test employed by the author is Jaffe's, which is applied as follows. The urine is mixed with an equal quantity of strong hydrochloric acid, by which means the indoxyl sulphate is decomposed and indoxyl liberated. A minute quantity of calcium hypochlorite is now added (the avoidance of excess is important), which oxidises the indoxyl with the production of indigo blue. If the urine be now shaken with chloroform, the indigo-blue goes into solution in the latter, and the depth of colour resulting is an index of the quantity of indoxyl present in the specimen. In normal health the colour is the faintest possible blue, or it may even be absent, but in the cases under notice a deep blue was generally obtained, so deep in some cases as to be almost black.

Dr. Townsend finds that this excessive indoxyl excretion, while universally present in acute melancholy, is almost invariably absent in acute mania; that the degree of excess appears to bear a regular proportion to the severity of the case, and that it disappears with the retrogression of the mental malady. He admits that excess of indoxyl may appear without any coexisting mental phenomena, a fact to be explained by the well-recognised variations in susceptibility to drugs or poisons evinced by different individuals. The neuropathic inheritance is thus presumed to include a diminished resistance to these abnormal products of albuminous metabolism.

Several cases in point are mentioned in support of the author's thesis. In all but one mental improvement was associated with a gradual diminution in the excess of indoxyl excretion, and the exception deserves record. The patient, a female, improved so greatly as regards her mental state that she was considered practically cured, the continued excess of indoxyl being regarded only as a curiosity. Shortly before her contemplated discharge she escaped and committed suicide, leaving a letter showing that her mental attitude was far other than her demeanour had led the authorities to believe.

The author's main deductions are as follows:—(1) That in depressed states indoxyl is excreted in excess; (2) That patients excreting indoxyl in excess exhibit symptoms and signs of toxæmia; (3) That in states of mental elation there is seldom any increase, the amount excreted being normal or less than normal; (4) That the more severe the mental attack the greater the excess of indoxyl; (5) That mental recovery is preceded by the reduction to normal of the amount of indoxyl excreted.

Treatment along these lines would naturally suggest reliance upon reputed intestinal disinfectants. Dr. Townsend has tried a number of them but without success. The best results are obtained from free purgation and, if it be possible, a diet entirely of milk.

There is undoubtedly a good deal to be said in favour of a toxic origin for certain of the insanities. We have good reason to know, for instance, that alcohol in large doses gives rise to a condition of temporary insanity, as do belladonna, cannabis indica, and a variety of other vegetable bodies. But that any one particular poison is the uniform cause of one particular group of mental disturbances is not proven, and Dr. Townsend very properly admits that in the case of indoxyl excess it is at present impossible to distinguish whether it stands to the mental state in the relation of cause or effect. When it is considered how many bacterial diseases, influenza, for example, or typhoid fever or diphtheria, may be the starting-point of an attack of mental depression, the suggestion is forced upon us that the neuropathic inheritance, if it is to be correlated with toxic processes at all, merely tends to direct the locality of their assault, instead of manifesting itself by an idiosyncrasy towards poisons of certain specific denominations.

[1] Jour. of Mental Sci., Jan. 1905.

